# Behavioural and electrophysiological correlates of lightness contrast and assimilation

**DOI:** 10.1007/s00221-021-06197-3

**Published:** 2021-08-26

**Authors:** Stephanie L. Acaster, Naira A. Taroyan, Alessandro Soranzo, John G. Reidy

**Affiliations:** Department of Psychology, Sociology and Politics, Faculty of Social Sciences and Humanities, Sheffield Hallam University, Sheffield, UK

**Keywords:** Lightness perception, Contrast, Assimilation, Event-related potentials, Reaction times

## Abstract

Lightness contrast and assimilation are opposite phenomena: in contrast grey targets appear darker when bordering bright rather than dark surfaces; in assimilation grey targets appear lighter when bordering bright rather than dark surfaces. The underlying neurophysiological mechanisms of these phenomena are not known. The aim of this study was to investigate the relationship between contrast and assimilation, and the timing and levels of perceptual and cognitive processing using combined behavioural and electrophysiological methods. Thirty undergraduate students (23 female, age range 18–48 years) participated in a forced-choice (grey target is lighter/darker than a comparison square) task, using stimuli designed such that the inducers were in two configurations (small and large) and two shades (white and black). The behavioural data (more consistent and faster responses) corroborated previous findings of stronger contrast effects with white inducers and stronger assimilation effects with black inducers. According to the Event-Related Potentials (ERP) results the mean amplitude was larger in conditions with less consistent and slower behavioural responses. Thus, with contrast responses P1 amplitude was larger with black than white inducers, and N1 amplitude was larger to assimilation than contrast when the configuration of the stimulus was held constant. These results suggest contrast may occur as early as P1 (~ 110 ms) and assimilation may occur later in N2 (~ 220 ms), whereas in some conditions, differences in ERPs associated with contrast vs assimilation may happen as early as in N1 (~ 170 m), in occipital and parietal cortical sites.

## Introduction

**Fig. 1:**
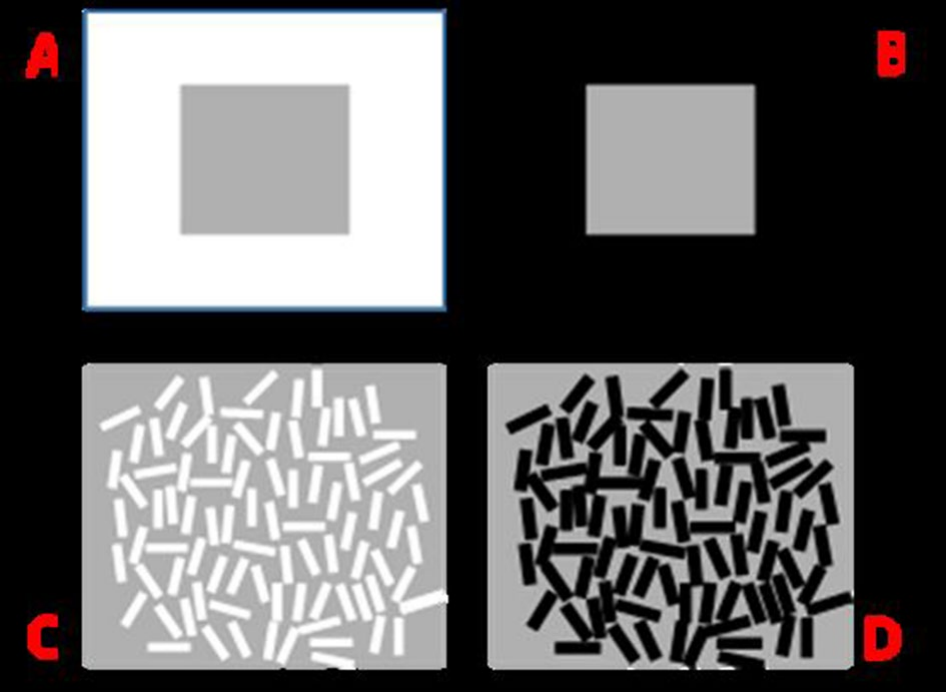
Examples of lightness contrast (**A** and **B**) and assimilation (**C** and **D**). The luminance of the grey is identical in all cases, but appears lighter in **B** and **C**

In lightness contrast, the perceptual quality of a surface appears to shift away from that of its neighbouring surface: a grey surface appears darker when it borders a light surface, and lighter when bordering a dark surface (see Fig. 1A, B; see also Kingdom 1999; Wade 1996). Conversely, in lightness assimilation, the perceptual quality of a surface appears to shift towards that of its neighbouring surface: a grey surface appears lighter when bordering a light surface and darker when bordering a dark surface (Fig. 1C, D; see also Soranzo et al. 2010; Soranzo et al. 2020). Given that contrast can be thought of as an effect which operates in the opposite direction to that of assimilation, the relationship between them presents an intriguing paradox in visual perception, whereby the same grey surfaces can produce different percepts.

While some authors stated that contrast and assimilation may be manifestations of a single underlying process (e.g., Kingdom 2011), others suggested that these are two completely distinct processes or (e.g., Kanizsa 1979). The psychological factors, relating to perceptual processing rather than the physical properties of the stimulus have also not been widely agreed upon (e.g., Agostini et al. 2014). Several branches of explanation exist which aim to account for the ‘shift’ between contrast and assimilation, spanning a range of ‘low-level’ physiological factors at the retinal level, to ‘higher-level’ perceptual/cognitive factors in the wider context of the image. For example, some low-level interpretations attribute both contrast and assimilation to lateral inhibition and neuronal spatial integration within centre-surround receptive fields (DeValois and DeValois 1975; Hurvich and Jameson 1966, 1974; Jameson and Hurvich 1975). Thus, Jameson and Hurvich (1975) suggested that in contrast, the differences between adjacent surfaces in the retinal image are enhanced as a result of spatial antagonism in centre-surround receptive fields, and in assimilation, the cell responds as though the colours are superimposed, making them appear more similar to one another. According to the Anchoring theory, instead, lightness contrast can be attributed to higher level cortical processing mechanisms, and assimilation to lower level retinal processing mechanisms (Gilchrist et al. 1999).

The importance of higher level processing in both phenomena has been increasingly recognised as more examples which cannot be accounted for by purely low-level mechanisms have been studied, such as ‘reversed’ contrast effects (e.g., Soranzo et al. 2010; Agostini et al. 2014). Thus, theories about the underlying factors have begun to take into account more information about the context. ‘Edge-integration’ theories propose that in lightness perception a ‘higher-level’ neural representation of the visual scene is produced by a physiological detection of local edge structure and spatial integration (Rudd 2010,2013), and lightness values are computed from a weighted sum of the responses of edge detector neurons in visual cortex.

An understanding of the neurophysiological basis of lightness contrast and assimilation would provide further crucial evidence in terms of delineating the types of underlying processing involved. Boyaci et al. (2007) reported activation in early visual regions related to context-dependent changes in lightness in response to the Craik–O’Brien–Cornsweet’ illusion, where two physically identical grey areas appear to differ in lightness (Cornsweet 1970), and independently of attention selection to the stimulus (Boyaci et al. 2010). Maertens et al. (2015) argued that perceptual interpretation of a surface strongly influences lightness assimilation which may require higher level cortical areas with sensitivity to perceptual organisation, such as V4; however, no neuroimaging data were reported.

According to Boucard et al. (2005), an fMRI signal of similar magnitude but delayed onset was recorded in the visual cortex (V1) in response to perceived changes in the brightness of a surface induced by variations in the luminance of the surrounding area compared to actual changes in surface luminance. The authors concluded that fMRI signals do not explicitly indicate the representation of brightness in the visual cortex, but that visual regions may be indirectly involved in surface brightness perception, and suggested further investigation of the temporal, as well as spatial, characteristics of brain activity associated with brightness perception. Pereverzeva and Murray (2008) also provided evidence to support a correlation between V1 fMRI activity and perceived lightness changes, in stimuli using modulation of the surrounding luminance to induce perceptual changes in the lightness of a target. Thus, some findings from fMRI research provide evidence to suggest that context-related changes in lightness perception are associated with early visual processing in V1. However, these findings do not give any information about potential differences or similarities between contrast and assimilation (as two types of lightness perception), nor do they indicate anything about the timing of the associated neural activity.

The ERP methodology offers high temporal resolution recording of brain activation and is a suitable tool in researching the time course and processing levels of neurophysiological mechanisms underlying contrast and assimilation. There has been little research so far in this area using ERP techniques. For example, McCourt and Foxe (2004) reported larger C1 (initial component of visual evoked potentials, VEP, around 70 ms post-stimulus-onset) amplitude with grey-on-white than grey-on-black targets at parieto-occipital sites. However, in this study contrast occurred in a major proportion of trials, as it was reported that grey patches presented on white were more likely to be judged as darker, and grey patches presented on black were more likely to be judged as lighter. Therefore, this evidence cannot be used to make a comparison between contrast and assimilation. In addition, although McCourt and Foxe focused on early brain activation, it remains of interest to examine later (after 100 ms) ERP components with regard to contrast and assimilation. This is particularly relevant, where further manipulations of the stimulus conditions, such as size of inducers and perceptual organisation of the stimulus, come into play, as these factors may be processed by different, perhaps higher level mechanisms than the colour (black vs white) of the surfaces bordering the grey under consideration. The early effect reported by McCourt and Foxe is arguably an index of the perceptual change, but their research does not show whether this effect may be modified by further stimulus manipulations. In addition, Sulykos and Czigler (2014) suggested that the perception of illusory changes in lightness differs from the perception of ‘real’, physically defined differences in luminance, and that these differences can be shown particularly in early (P1 and N1) ERP components. However, lightness contrast and assimilation have not been directly compared and studied together using both behavioural and ERP methods, in the way that they have been in the purely behavioural and psychophysical literature.

The current project aimed to study the electrophysiological and behavioural correlates of contrast and assimilation and give an insight into the time course of the underlying neural processing. To the best of our knowledge, there is no neurophysiological research on lightness contrast and assimilation in parallel, hence this study aimed to contribute to existing knowledge of the two phenomena and form a bridge from the extensive behavioural/psychophysical literature into an understanding of the associated neural processing. The value of using ERP methods to study contrast and assimilation is that they may allow us to determine the importance of later, higher level processes to the generation of these differing effects. We employed a novel paradigm specifically designed for ERP research that involved a 2-alternative forced-choice (2AFC) task, where the stimulus was presented alongside the comparison square and the participant was required to decide whether it was lighter or darker than the comparison square. We aimed to find out whether the colour (white, black) or configuration (small, large) of the inducers would affect the perception and how these will be reflected in the behavioural and ERP correlates.

1. Based on previous psychophysical literature (e.g., Soranzo et al. 2010) we too expected to demonstrate contrast and assimilation responses with white and black inducers. Moreover, consistent with Soranzo et al. (2020), we expected more contrast responses (and faster RTs) to stimuli with white large than black large inducers, and more assimilation responses to stimuli with black small inducers than white small inducers.
2. As in previous studies (e.g., McCourt and Foxe 2004) we too expected both phenomena to result in activity in occipital and parietal areas, though given the exploratory nature of the current research the relative levels of activity for each phenomenon are uncertain. As suggested above, such activity, if observed, may enable us to determine the involvement of higher mental processes in these phenomena.
3. Given the findings from Sulykos and Czigler (2014) we also hypothesised that contrast and assimilation would produce differences in components P1 or later. We suggest that if such differences do arise in P1 and later ERP components then this suggests high-level cortical processes rather than low-level retinal processes may be responsible for the different phenomena.
4. Finally, we hypothesised that the strength of a contrast (or assimilation) effect would correlate with the ERP amplitude in the components associated with each effect, such that where a stimulus elicits more contrast responses, the amplitude may be greater than a stimulus which elicits fewer contrast responses. Such correlations would strengthen the evidence that these components are critical in the generation of contrast or assimilation.

Although, this study is exploratory in nature it is possible that any differences observed in the ERP analyses may hint at underlying processing differences between contrast an assimilation.

## Methods

### Participants

Thirty participants (7 males) in the age range 18–48 years (*M* = 23.57, SD = 8.41) were recruited in exchange for either undergraduate research participation credits or a high street voucher. They were all right-handed (confirmed using a revised version of the Edinburgh Handedness Inventory; Oldfield 1971; Veale 2014), with normal or corrected-to-normal vision and no history of neurological problems/disorders. One participant was excluded from analysis for not completing the whole task, another was excluded for giving too many incorrect responses to ‘catch’ trials (i.e., those with a physical luminance difference between target and comparison square, designed to check participants’ attention to the task). The study was approved by the local ethics committee of the Psychology Department at Sheffield Hallam University, and written informed consent was obtained from all participants before the testing begun.

### Stimuli and task

**Fig. 2:**
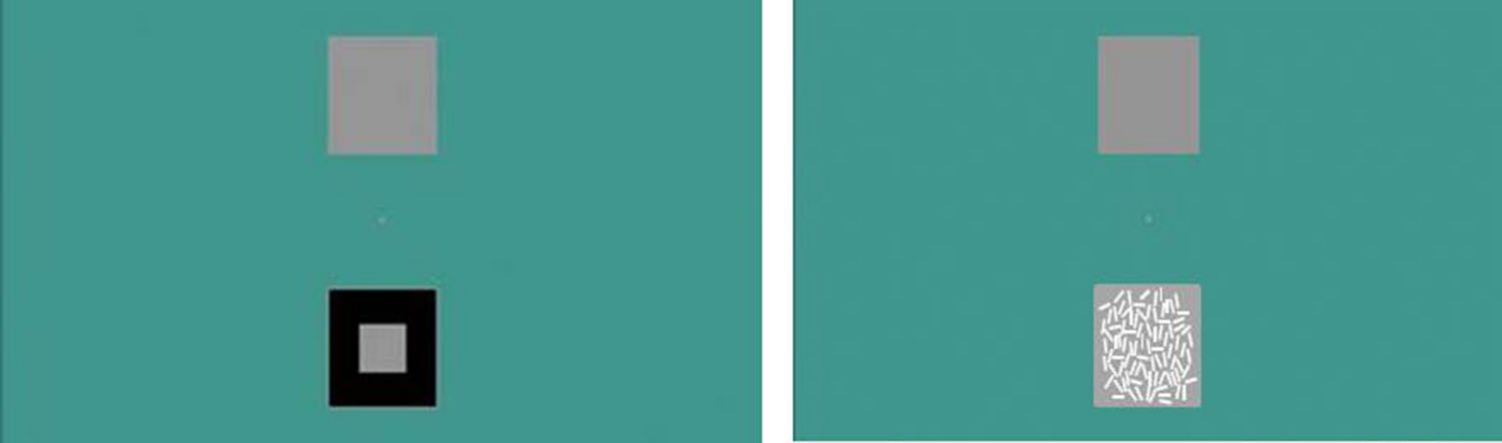
Examples of the stimulus (bottom left—contrast, bottom right—assimilation) and the comparison square (top), as presented in the tasks. The participants viewed a fixation cross, then one of these screens to present the stimulus, followed by a blank screen inter-stimulus interval

Preliminary methodological studies were carried out to design the stimuli based on previous research (e.g., Soranzo et al. 2010, 2020). The aim was to maximise the rate of contrast responses (to contrast-inducing stimuli) and assimilation responses (to assimilation-inducing stimuli) for a robust investigation of the ERP responses associated with contrast and assimilation. The ‘large’ inducers designed to elicit contrast consisted of a 5 × 5 cm square, containing a smaller, 2 × 2 cm square in the centre, whereas the ‘small’ inducers designed to elicit assimilation consisted of a 5 × 5 cm square target scattered with 88 small rectangles (0.75 × 0.31 cm) accumulating to a total surface area of 20.46 cm^2^ approximately matching the inducer surface area of 21 cm^2^ in the contrast stimuli (see Fig. 2).

The stimuli were presented on a CRT screen monitor (NEC MultiSync FP2141sb) using E-prime (version 2.0.10.353; Psychology Software Tools Inc.). The CRT screen was chosen (rather than an LCD monitor) as it offers a more uniform luminance and colour of the display regardless of viewing angle. Each stimulus was randomly displayed either at the top or bottom of the screen and in equal numbers, along with a blank grey ‘comparison’ square of identical physical luminance to the ‘target’ grey, as depicted in Fig. 2. The luminance values of surfaces measured with a photometer were 95.78 cd/m^2^ for the white, 0.55 cd/m^2^ for the black and 29.89 cd/m^2^ for the grey inducers. These unequal luminance ratios were selected after pilot experiments to elicit the highest possible rate of contrast and assimilation response to measure the corresponding ERPs.

The remainder of the screen was set to a blue–green hue of the same measured luminance as the grey to minimise the likelihood of the responses being influenced by the luminance of the background.

Participants were seated at a 57 cm distance from the screen to ensure that 1 cm on the screen corresponded to 1 degree of visual angle. They were presented with each condition 80 times (320 in total: 80 × 4 stimulus conditions) along with 96 ‘catch’ trials distributed randomly amongst the experimental stimuli (there was generally a high level of correct responses on most of these catch trials, *M* = 91.2%, SD = 4.94) Unlike many of psychophysical studies that run only few repetitions of each condition, averaging ERPs from the EEG recording requires a large number of repetitions per condition to obtain a good signal-to-noise ratio, and requires responses that can be categorised and collected at a specific point in time. Hence, the 2AFC task was chosen over alternatives, such as a matching or adjustment task. Participants’ responses were recorded in a 2AFC task paradigm and categorised as contrast or assimilation, as shown in Table 1. If a participant did not respond on a particular trial, this was counted as a ‘miss’ and not taken as either type of response. In half of the trials, participants were asked to indicate which grey square was the darkest; and in the other half of trials, they were required to indicate which grey square was the lightest. The order of these instructions was counterbalanced among participants.

**Table 1:** Categorisation of responses for the 2AFC task

Inducers	Response (judging the target relative to the comparison square)	Categorisation of response
Black	Target is lighter than comparison	Contrast
	Target is darker than comparison	Assimilation
White	Target is lighter than comparison	Assimilation
	Target is darker than comparison	Contrast

### Procedure

After completing the consent form and handedness questionnaire, participants were then set up with EEG recording electrode cap. The instructions for their task were given verbally, as well as being presented on the screen. Participants were requested to indicate which one between the top or bottom of the screen was the lighter or darker grey, according to the experimental condition. They were then required to press the upper button of the response box with the right index finger if their chosen lighter/darker grey was presented at the top of the screen and the lower button with right middle finger if their chosen grey was displayed at the bottom of the screen.

In each trial, a fixation cross (500 ms) was presented, followed by presentation of the stimulus (3000 ms), which in turn was followed by a variable inter-stimulus interval (blank screen), of between 500 and 1500 ms. This is often used in ERP studies to minimise the effect of a potential source of artefactual activity from anticipatory activity which can occur during a cycle of identical timings between stimuli. Participants were instructed to look at the fixation cross, and refrain from making eye movements as much as possible (to reduce EOG artefacts). The seated distance from the screen allowed both the upper and lower positions to be seen with minimal-no movement.

Participants were asked to refrain from blinks during the stimulus presentation and other bodily movements to minimise ocular and other movement-based artefacts. They completed a short series of practice trials to familiarise themselves with the task. The main task was divided equally into four blocks of approximately 5 min each, with three short breaks. Each block, therefore, contained 80 experimental stimuli (20 × 4 conditions) and 24 ‘catch’ trials. The task lasted about 35–40 min.

### Data acquisition

The EEG recording system consisted of a 128 Ag/AgCl electrode Waveguard EEG cap, with the electrodes arranged according to the five percent electrode system (Oostenveld and Praamstra 2001)—an extension of the traditional 10/20 system (Jasper 1958). The higher density allows an improved signal-to-noise ratio for electrode clusters compared to a 64-electrode system (Dien 1998). Electrode impedances were adjusted to below 5kΩ using electrode gel.

The EEG data were recorded within a range of 0.016–200 Hz with 512 Hz sampling rate, via a cascaded setup using two linked 64-channel amplifiers, through a SynFi fibre-to-USB converter. The recording software used was ASAlab 4.7.12 (Advanced Source Analysis laboratory; www.ant-neuro.com). Time-locked to the stimulus onset EEG waveforms and behavioural data, i.e., the type of response given (‘contrast’ vs ‘assimilation’) and reaction time (RT) from E-Prime, were transmitted to ASAlab via amplifiers.

### Data filtering and artefact removal

EEG data were processed for each participant prior to further analysis. First, the recorded data were re-referenced to an average reference (Dien 1998). The high-pass filter cutoff was set at 0.01 Hz, 12 dB/octave (1–2 Hz automatically removed by the software), to remove slow voltage shift artefacts caused by drifts in electrode impedance or sweating with minimal distortion (Luck 2014; Tanner et al. 2016). The low-pass filter cutoff was set at 40 Hz, 24 dB/octave, to filter out muscle movement and line frequency noise such as that from AC electrical devices. Artefact detection was then carried out by visual inspection and manual marking of large ocular artefacts (i.e., eye blinks), then by automated artefact detection to find recorded activity greater than ± 70 μV to identify smaller artefacts, such as eye moments. The ASA artefact correction procedure used a PCA method to separate signals and artefacts (Ille et al. 2002).

### Data analysis

Mean RTs and number of responses for each condition were calculated for each participant. ERPs were derived from 100 ms pre- to 1000 ms post-stimulus EEG epochs in response to contrast or assimilation. Any segments with artefacts that could not be corrected were rejected and the ERPs computed by averaging all the remaining trials, separately for contrast and assimilation responses. Baseline correction was then performed by subtracting the average pre-stimulus voltage from the waveforms.

**Fig. 3:**
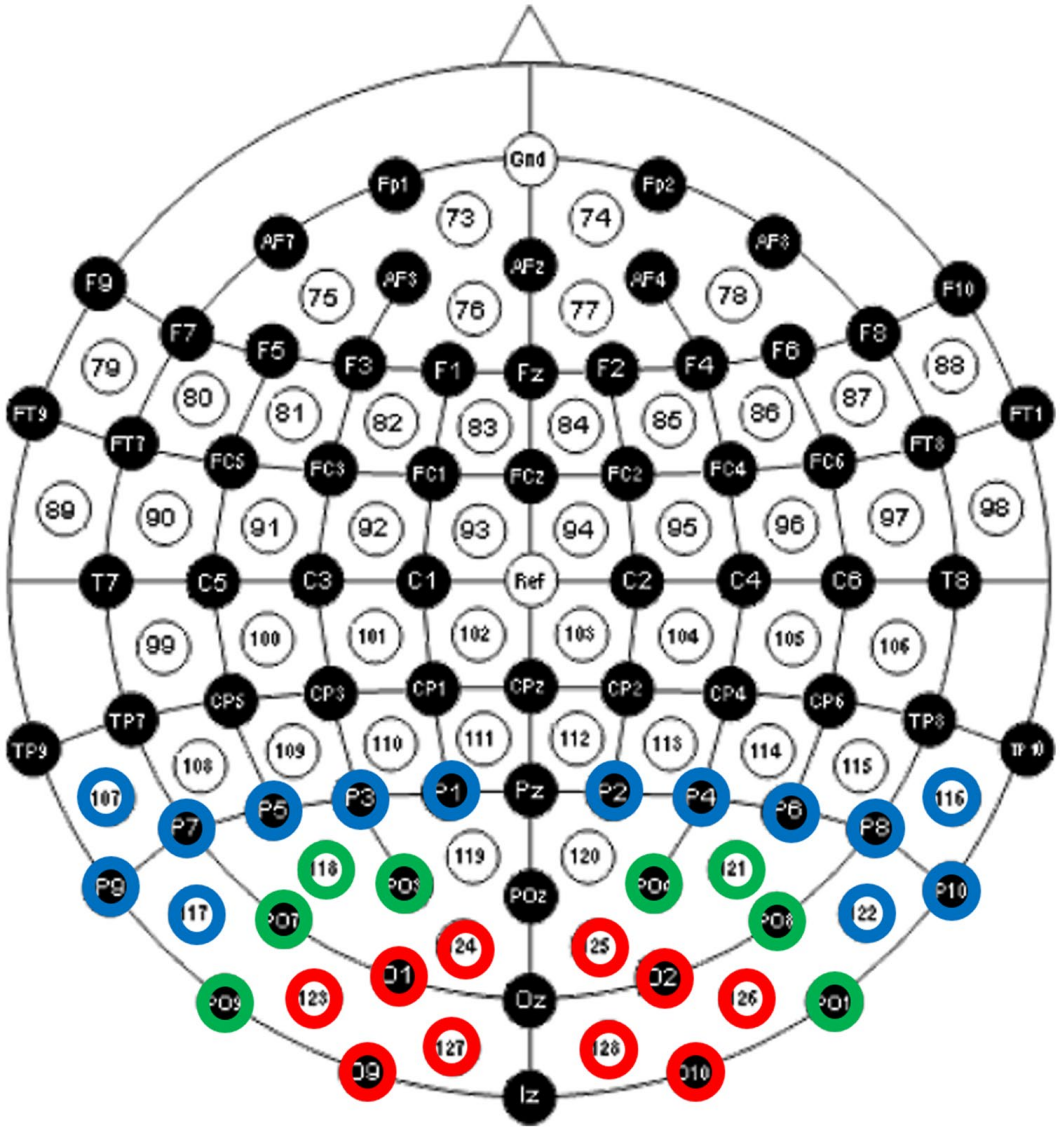
Electrode clusters in their relative positions. The electrodes included in the ‘occipital’ cluster are circled in red; ‘occipital–parietal’ in green; and ‘parietal’ in blue

The occipital (O), occipital–parietal (OP) and parietal (P) regions in the left and right hemispheres were selected for further analysis, as they are associated with visual processing and were highlighted in previous lightness-related ERP research (e.g., McCourt and Foxe 2004). In addition, visual inspection of the data indicated clear and larger ERP peaks in these areas. The waveforms were separated into three clusters of neighbouring electrode sites in each hemisphere (see Fig. 3), to optimise signal to noise ratio and increase statistical power (Dien 2017; Oken and Chiappa 1986).

Collapsed grand average ERP waveforms from all participants, regardless of condition/response type were also constructed (see Luck and Gaspelin 2017; Handy 2005) and used to define measurement windows for the peaks/components to be analysed, by inspecting the overall shape of the waveforms, and focusing upon components which are consistently present and prominent in visual paradigms, such as P1 and N1. Measurement windows (ideally > 50 ms when using mean amplitude, according to Luck 2014) for P1 (70–120 ms), N1 (130–180 ms), and N2 (190–240 ms) were selected based upon visual inspection of the individual waveforms, as well as a ‘collapsed average’ localiser. The mean amplitude for each ERP component and each participant was measured in specified windows and used for further analysis, as was the 50% fractional area latency, i.e., the latency which lies at the mid-point of the total area under the waveform within the measurement window. Using mean amplitude ensures that the ERP component is measured across the same latency range in each condition, rather than at a single point, which can be affected by high-frequency noise (for review see Picton et al. 2000; Luck and Gaspelin 2017). Mean amplitude is also a more legitimate measurement to use in cases where waveforms with differing noise levels, or those based on different numbers of trials are being compared. This is particularly relevant to some of the comparisons in this work, for example when comparing waveforms of sub-groups of participants based on their behavioural responses (which leads to unequal *n* in the two groups, ergo a difference in the number of trials included in the two waveforms). Similarly, peak latency does not take into account the shape or distribution of the waveform, can easily be distorted, and is particularly sensitive to noise. Fractional area latency is less sensitive to noise and is a more rigorous way of using the area contained by a section of the waveform to estimate the ‘midpoint’ latency, thereby taking into account more of the information provided by the waveform than a single peak point (Woodman 2010). This finds the latency point at which the area contained under a section of the waveform can be split into two equal-area parts.

**Fig. 4:**
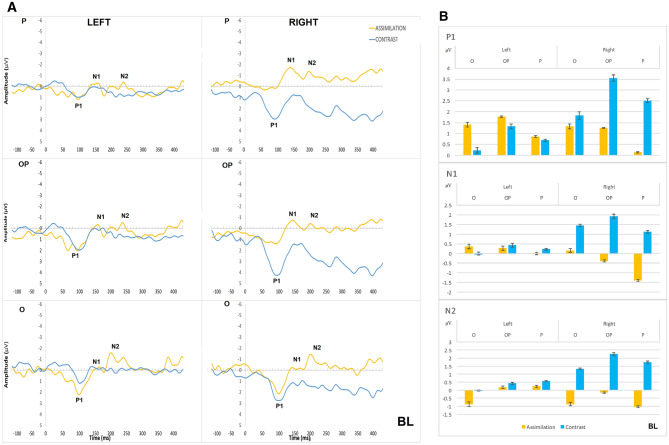
**A** Grand average ERP waveforms in left and right parietal (P; top), occipital–parietal (OP; middle) and occipital (O; bottom) sites for contrast (in blue) and assimilation responses (in yellow) in BL condition; **B** Mean amplitude per condition, hemisphere, and cluster for the time periods of P1, N1, and N2 in BL condition. Error bars represent standard errors

**Fig. 5:**
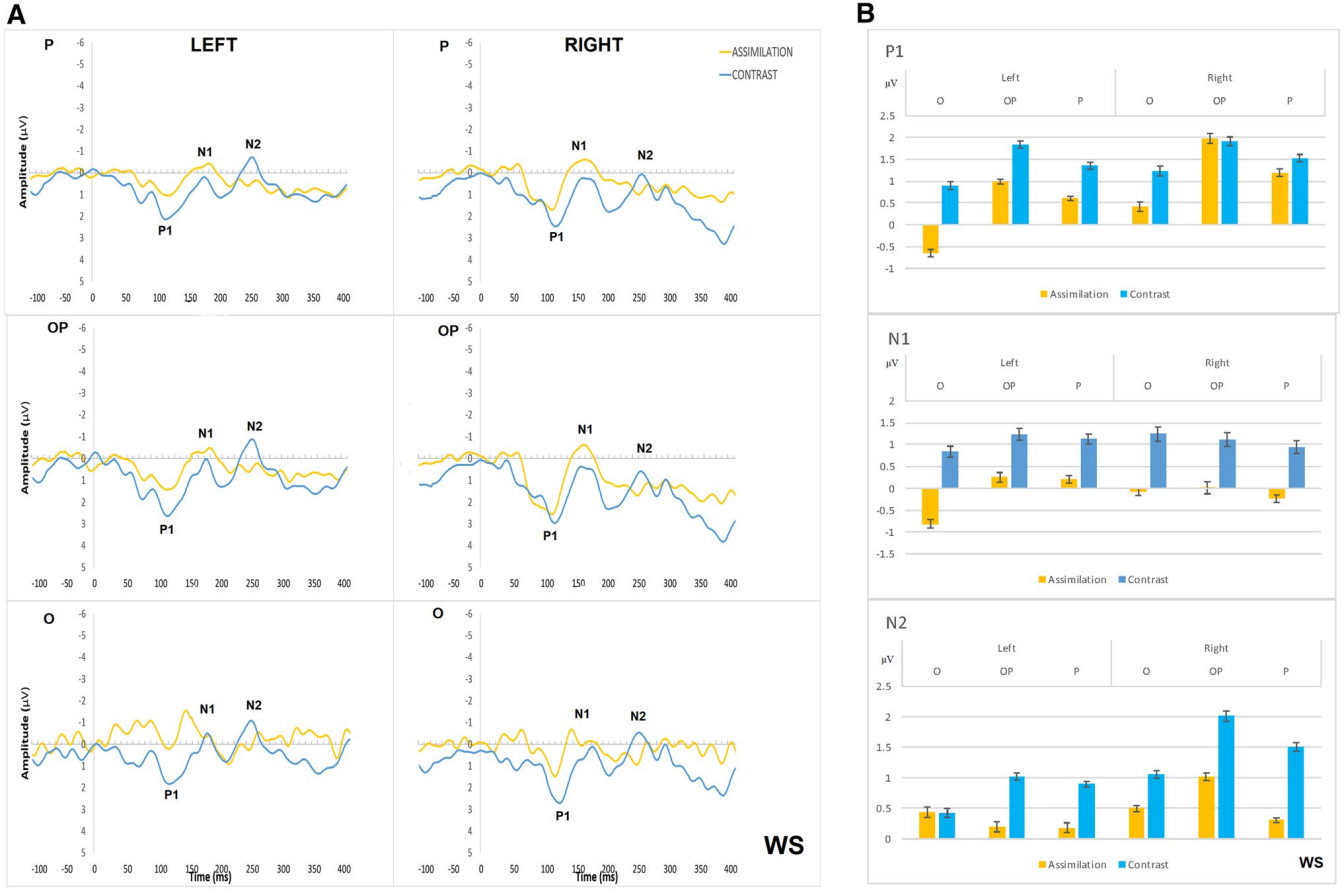
**A** Grand average ERP waveforms in left and right parietal (P; top), occipital–parietal (OP; middle) and occipital (O; bottom) sites for contrast (in blue) and assimilation responses (in yellow) in WS condition; **B** Mean amplitude per condition, hemisphere, and cluster for the time periods of P1, N1, and N2 in WS condition

**Fig. 6:**
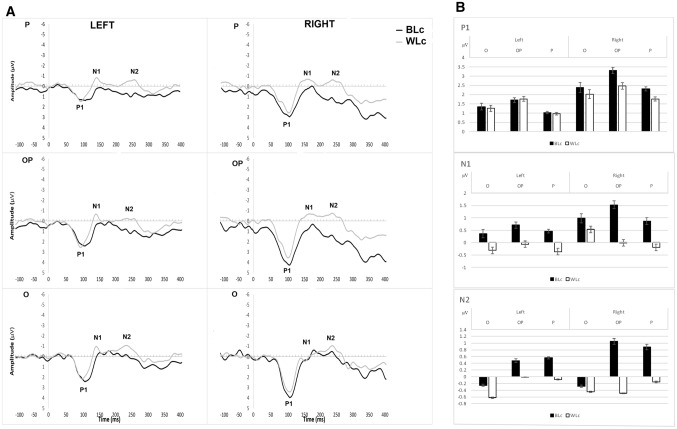
**A** Grand average ERP waveforms in the left and right parietal (P; top), occipital–parietal (OP; middle) and occipital (O; bottom) sites for contrast responses to BL (in black) and WL (in white) conditions; **B** Mean amplitude per condition (BL, WL), hemisphere, and cluster for the time periods of P1, N1, and N2

**Fig. 7:**
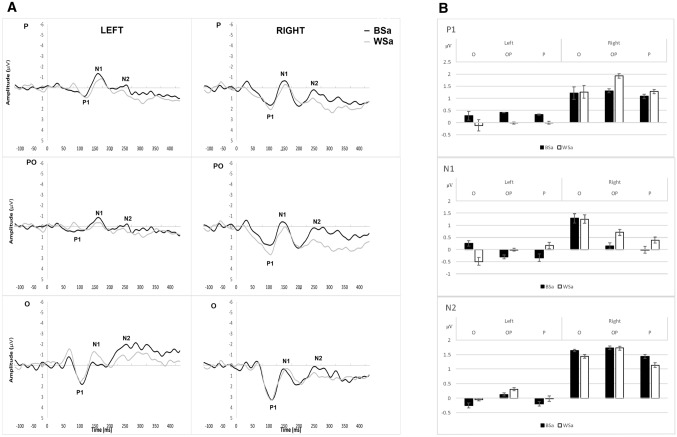
**A** Grand average ERP waveforms in the left and right parietal (P; top), occipital–parietal (OP; middle) and occipital (O; bottom) sites, for assimilation in BS (in black) and WS (in white) conditions; **B** Mean amplitude per condition (BS, WS), hemisphere, and cluster for the time periods of P1, N1, and N2

Therefore, the grand average waveforms (Figs. 4A, 5A, 6A, and 7A), represent only approximate information on ERP components and were used to guide the selection of measurement windows for individual participant ERPs. The mean amplitude and latency of the ERP components were then derived from these individual participants data using *jackknife* approach (see below for brief explanation and advantages) and used in statistical analysis (group means shown in Figs. 4B, 5B, 6B, and 7B).

### Statistical analysis

For the behavioural data, repeated measures analyses of variance (ANOVAs) were carried out separately for the number of responses and RTs with two factors: response type (contrast or assimilation) and condition (referred to in terms of the properties of inducers—‘Black Large’ (BL), ‘Black Small’ (BS), ‘White Large’ (WL), and ‘White Small’ (WS).

Statistical analysis of ERP components’ mean amplitude and latency was carried out via a four factor ANOVA: response type (contrast and assimilation), condition (BL, BS, WL and WS), hemisphere (Left and Right), and electrode cluster (O, OP and P). More detail on each individual statistical analysis is given in the Results section. The Greenhouse–Geisser correction has been used in reporting ANOVAs when there were more than two levels of a factor (Luck 2014, p 320). This is relevant when comparing more than two conditions or more than two clusters of electrodes in an analysis, because there is likely to be heterogeneity of variance with three or more electrode areas, due to the tendency for covariance to be lower for a pair of electrode areas situated a distance apart, than for a pair of electrode areas situated near to one another. The Bonferroni adjustment for multiple comparisons was made during post-hoc tests following ANOVAs.

A *jackknife* approach has been used in this study as it increases statistical power while retaining Type 1 error rates (Kiesel et al. 2008). Traditional ERP analysis constructs a grand average (composed of all participants’ data) waveform in each condition to describe the data but uses the amplitude and latency values of components calculated from the ERPs of each individual participant. In doing so, the improved signal-to-noise ratio of the grand average waveforms is not used in the analysis, unlike the jackknife method (e.g., Abdi and Williams 2013; Kiesel et al. 2008; Miller et al. 1998). It involves the computation of ‘leave-one-out’ grand averages, whereby each participant is replaced by a ‘subaverage’ of the other n-1 participants within the same group/condition. The resulting ‘subaverage’ waveforms will have a substantially larger signal-to-noise ratio compared to an individual participant’s waveform (Miller et al. 1998), thereby reducing within-participant variance (Abdi and Williams 2013) and potentially minimising irrelevant between-participant effects such as those arising due to differences in mood or arousal (Kornmeier et al. 2016). This is particularly useful in cases, where some conditions may be more affected by poor signal-to-noise ratio due to a smaller number of trials/participants. Since the jackknife technique is based on averaged data, the variance of the set of subaverage scores is smaller than the variance of the original scores would be (Ulrich and Miller 2001). Therefore, adjustments to the computations of standard statistical tests have been developed: Miller et al. (1998) provided an adjusted equation for the standard error, and Ulrich and Miller (2001) derived an adjustment of *F* values in ANOVA for factorial designs for *t* tests, by dividing the *t* value by (*N* − 1); for *F* tests, dividing the *F* ratio by (*N* − 1)^2^ (Luck 2014). An adjustment was also made to the effect sizes, calculating the adjusted *η*^2^ = 1/(1 + 1/*F*_*adj*_ × df_error_/df) for ANOVAs with adjusted *F* values.

## Results

### Categorical representation of response and data pre-processing

For each condition, a total (out of a possible 80 trials per participant) was calculated for each type of response. None of these total values fell outside of 3 standard deviations from their respective condition mean, so were not considered to be outliers. Out of two types of responses, i.e., contrast and assimilation, 57%, 23%, 83% and 54% were contrast perceived responses in BL, BS, WL and WS conditions, respectively, with fewer than 1% of missed trials across all conditions.

There was a significant main effect of inducer colour on the number of contrast responses, *F*(1,27) = 21.44, *p* < 0.001, *η*_*p*_^2^ = 0.44, whereby there was a lower number of contrast responses in conditions with black inducers than in conditions with white inducers. There was also a significant effect of the configuration of inducers, *F*(1,27) = 80.86, *p* < 0.001, *η*_*p*_^2^ = 0.75, whereby there was a higher number of contrast responses in conditions with large than with small inducers. These results confirm that overall, the stimuli and the task chosen do still produce the contrast and assimilation effects that would be expected with equivalent stimuli in another paradigm (e.g., matching or adjustment). The interaction between inducer colour and stimulus configuration was not significant, *F*(1,27) = 0.712, *p* = 0.406, *η*_*p*_^2^ = 0.03. The WL condition elicited the most contrast and the BS—the most assimilation responses.

Planned one-tailed repeated measures *t* tests were carried out within each condition, to check whether the contrast and assimilation effects were consistent for a given stimulus, as it could be seen from the data set that there was variability in participants’ responses, i.e., some participants gave contrast responses every time in the condition with black, large inducers, whereas others gave mixed contrast/assimilation responses over repeated trials. These showed no significant difference between contrast and assimilation in the BL, *t*(27) = 1.30, *p* = 0.207, or the WS condition, *t*(27) = 0.79, *p* = 0.438, but more assimilation than contrast responses in the BS, *t*(27) = 5.67, *p* < 0.00, and more contrast than assimilation responses in the WL condition, *t*(27) = 7.54, *p* < 0.001. This is consistent with the perception of assimilation being stronger with black inducers than with white inducers (e.g., de Weert and Spillman 1995; Soranzo et al. 2010) and the perception of contrast being stronger with white inducers than with black inducers (e.g., Beck 1966) but not consistent with Economou et al.’s (2007) finding that contrast effects are stronger with black inducers.

The results showed that some participants gave consistent, i.e., mostly contrast or mostly assimilation, responses within a condition, whereas others gave more mixed responses. A criterion of whether a participant’s distribution of responses was not simply chance-level performance was set at 53 (out of 80) responses, this was deduced by comparing a hypothetical data set with a set of evenly split responses. The hypothetical data set differed significantly from the evenly split (40–40) responses when its distribution was 53–27 (*χ*^2^(1) = 4.39, *p* = 0.037). On this basis, where ≥ 53 responses out of 80 trials were contrast responses, the participant was taken to have given consistent contrast responses for that condition (likewise for assimilation). Where responses were more mixed (i.e., between 28 and 52 responses of each type within the same condition) this was deemed not to differ significantly from a ‘chance-level’ performance, and therefore, the participant was categorised as having given ‘inconsistent’ responses in that condition. A Chi-square analysis showed the distribution of frequencies within the response types across conditions deviate significantly from what would be expected by chance (*χ*^2^(6) = 40.28, *p* < 0.001).

The categorisation of participants in each condition according to the consistency of their responses was important for the ERP analysis for several reasons. First, there was uncertainty as to whether inconsistent responders perceived the stimuli in the same way as consistent responders as they were more likely to be guessing or unsure of their response. For consistent responders, a larger number of trials were included in the ERP averages which is likely to have resulted in a better signal-to-noise ratio. Taking forward the cases, where response type was classified as being consistent presents a difficulty in analysing across all four conditions, as a participant giving consistent responses in a particular condition may give inconsistent responses in other conditions. Therefore, certain comparisons cannot be conducted within subjects (i.e., those where a participant does not give consistent responses across more than one condition); however, it enables between-subjects comparisons to be made between different categories of response.

### ERP and behavioural data

An overall analysis, including all participants, was carried out to compare the ERPs associated with contrast responses vs those associated with assimilation responses, regardless of condition. Thus, for this analysis, conditions were not entered as a variable, and the independent variables were simply response type (i.e., contrast and assimilation), and hemisphere and cluster. For the amplitude of P1, N1, and N2, there were no significant three-way interactions (*p* values > 0.7), nor any significant two-way interactions (all *p* values > 0.4) or main effects (all *p* values > 0.7). Similarly, for the fractional latency of P1, N1 and N2, there were no significant three-way interactions (*p* values > 0.6), nor any significant two-way interactions (all *p* values > 0.6) or main effects (all *p* values > 0.4).

### Comparing contrast and assimilation within the same condition

In the BL condition, 13 participants gave consistent contrast responses and 7 participants gave consistent assimilation responses, whereas in the WS condition, 12 participants gave consistent contrast responses and 7 participants gave consistent assimilation responses. The first two comparisons were done to compare ERPs to the two types of response while holding constant the condition: (1) contrast vs assimilation within the BL condition and (2) contrast vs assimilation within the WS condition. Initially, the split between contrast and assimilation responses in these two conditions was a somewhat unexpected result, as stimuli were intended to elicit either contrast or assimilation responses, not both. However, the emergence of the different responses allows the comparison of ERPs to two different perceptions of the same physical stimulus.

### Contrast vs assimilation responses within the BL condition (response type as a between-groups factor)

The difference in RT between those consistently giving contrast responses and those consistently giving assimilation responses was not statistically significant, *U* = 16, *z* = − 1.71, *p* = 0.088. For the mean amplitude of P1, N1 and N2, the three-way interactions between response type (contrast vs assimilation), hemisphere (left vs right), and electrode cluster were not significant (largest *p* = 0.170). Overall, the amplitude of the ERP components was larger and their latency longer in parietal areas compared to occipital and occipital–parietal (all *p* values < 0.001). Although the difference between contrast and assimilation appears to be more pronounced in the right hemisphere than in the left (see Fig. 4), no other significant main effects were found. There were significant hemisphere x electrode cluster interactions with a shorter N1 latency (*p* = *0.008*) and greater N2 amplitude (*p* < 0.001) in the right than the left occipital cluster.

### Contrast vs assimilation within the WS condition (response type as a between-groups factor)

**Fig. 8:**
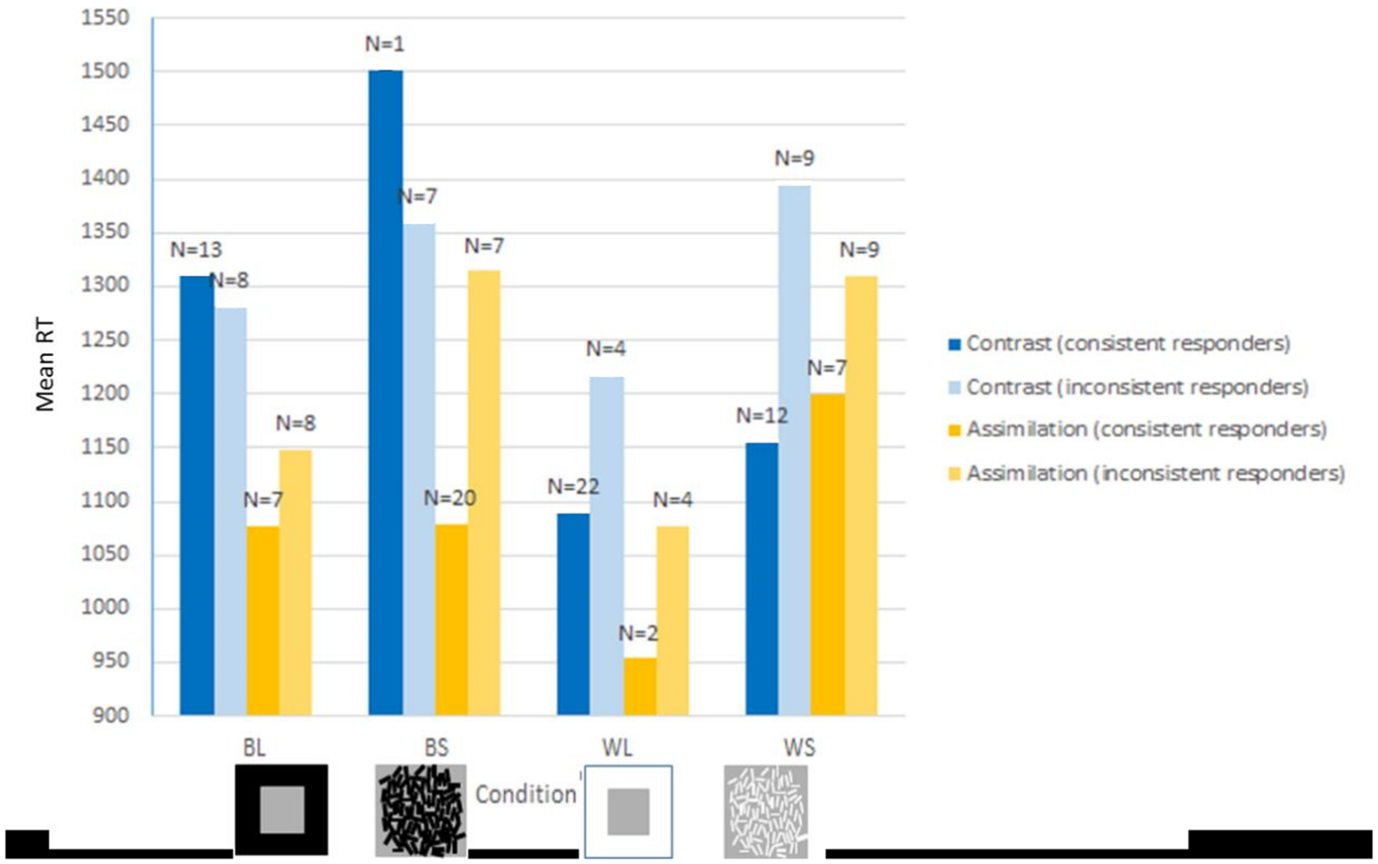
Mean RT for each type of response within each condition for consistent (either contrast or assimilation) or inconsistent (both contrast and assimilation) responders and separated by response type. The inconsistent responders are, therefore, included in two columns to compare the RTs associated with either of the two responses given. The number of responders is indicated at the top of each column

In this condition, there was no significant difference in RT between those consistently giving contrast responses and those consistently giving assimilation responses, *U* = 34, *z* = − 0.41, *p* = 0.684. However, the mean RTs were slightly higher for assimilation than for contrast (See Fig. 8).

For the *mean amplitude of P1*, there were significant main effects of hemisphere [*F*(1, 13) = 17.13, *p* = 0.001, *η*_*p*_^2^ = 0.57] and cluster [*F*(1.65, 21.38) = 37.25, *p* < 0.001, *η*_*p*_^2^ = 0.74], with the right hemisphere showing greater P1 amplitude than the left in occipital (*p* < 0.001), occipital–parietal (*p* = 0.008), and parietal (*p* = 0.006) areas (see Fig. 5)*.*

For the *mean amplitude of N1*, the three-way interaction between response type, hemisphere, and electrode cluster was significant [*F*(1.85, 24.03) = 4.57, *p* = 0.020, *η*_*p*_^2^ = 0.260]. Post-hoc two-way ANOVAs showed that the response type x electrode cluster interaction was significant in the left hemisphere [*F*(1.74, 22.67) = 4.00, *p* = 0.031], whereby the N1 amplitude was greater for assimilation responses than for contrast responses in the left occipital area [*t*(13) = 2.26, *p* = 0.042] only (see Fig. 5). The right hemisphere did not show significant differences between assimilation and contrast responses. There was also a significant main effect of electrode cluster [*F*(1.48, 19.26) = 51.60, *p* < 0.001], with post-hoc comparisons showing a greater N1 amplitude in the parietal cluster compared to the occipital (*p* < 0.001) and occipital–parietal clusters (*p* < 0.001) in both hemispheres. The mean amplitude of N2 was also greater in parietal than occipital (*p* < 0.001) and occipital–parietal clusters (*p* < 0.001) in both hemispheres (see Fig. 5).

### Comparing different conditions, where the same response type is observed

The following ERP comparisons were made to hold constant the type of response and the stimulus configuration, with the comparison being between the inducer colour: (1) contrast responses between the BL and the WL conditions, and (2) assimilation responses between the BS and the WS conditions; and the stimulus configuration: (3) assimilation responses between the BL and the BS condition, (4) contrast responses between the WL and the WS condition.

### Comparison of contrast responses of differing strengths: BL vs WL (All factors within-groups)

Of the participants who consistently gave contrast responses in both the BL and WL conditions, the contrast responses to WL were significantly faster than BL, *z* = − 2.67, *p* = 0.008 (see Fig. 8).

For the mean amplitude of P1, there was a significant three-way interaction between condition, hemisphere, and cluster [*F*_*adj*_(1.72, 13.75) = 5.98, *p* = 0.029, *η*^2^ = 0.43]. This was followed-up by two post hoc 2 (condition) × 3 (cluster) ANOVAs, for each hemisphere separately, which showed P1 mean amplitude was larger in the BL than the WL condition [*F*_*adj*_(1, 8) = 9.81, *p* = 0.001, *η*^2^ = 0.55] in the right occipital–parietal cluster only (*t*_*adj*_ = 3.07, *p* = 0.015) (see Fig. 6). For the fractional latency of P1, N1, and N2, there were no significant three-way interactions (*p* values > 0.8) nor any significant two-way interactions (all *p* values > 0.6) or main effects (all *p* values > 0.1).

### Assimilation responses in the BS and the WS conditions (All factors within-groups)

The RTs were delayed in WS compared to BS condition, however, not significantly, *z* = − 1.21, *p* = 0.225 (see Fig. 8).

For the *mean amplitude of P1 and N1* components, no significant interactions or main effects were found (all *p* values > 0.1), although their amplitude seemed larger in the right compared to the left hemisphere (see Fig. 7). For the *mean amplitude of N2*, there was a significant three-way interaction between condition, hemisphere, and cluster [*F*_*adj*_(1.53, 6.12) = 7.00; *p* = 0.038, *η*^2^ = 0.64]. This was followed-up by three post hoc 2 (condition) × 2 (hemisphere) ANOVAs which showed a significant two-way interaction between condition and hemisphere [*F*_*adj*_(1, 4) = 12.45; *p* = 0.002, *η*^2^ = 0.76] in the occipital cluster only. Post-hoc two-tailed paired *t* tests did not show any significant differences between the left and right hemisphere in the Black Small [*t*_*adj*_(4) = 1.56, *p* = 0.193] nor the White Small [*t*_*adj*_(4) = 1.13, *p* = 0.323] conditions. There were also no significant differences found between BS and WS in the left [*t*_*adj*_(4) = 1.47, *p* = 0.216] or right [*t*_*adj*_(4) = 0.50, *p* = 0.645] hemispheres. The lack of significant post-hoc effects in spite of a significant interaction may be a result of small sample size in this case. However, the N2 mean amplitude was more negative in the left than in the right occipital area; and the mean amplitude being greater for White Small than for Black Small in both hemispheres. For the fractional latency of P1, N1, and N2, there were no significant three-way interactions (*p* values > 0.4) nor any significant two-way interactions (all *p* values > 0.2) or main effects (all *p* values > 0.1).

### No significant results were found for the assimilation responses in the BL and the BS or the contrast responses in the WL and the WS conditions

In summary, a significantly greater N1 amplitude for assimilation responses than for contrast responses was found in the left occipital area in the WS condition. This greater N1 amplitude was associated with a weaker assimilation effect (slower and less consistent behavioural responses). A significantly larger P1 amplitude was found with contrast responses in the BL condition compared to the WL condition in the right occipital–parietal cluster. This larger P1 amplitude was associated with a weaker contrast effect (slower and less consistent responses). A greater N2 amplitude was found with assimilation responses in the WS condition compared to the BS condition in both hemispheres. For both contrast and assimilation, ERP amplitudes were generally larger in the right hemisphere compared to the left, and in the parietal compared to the occipital and occipital–parietal areas.

## Discussion

### Behavioural findings (hypothesis 1)

In this study we aimed to identify the time course of processing associated with lightness contrast and assimilation using a combined behavioural and ERP technique. In examining hypothesis 1, behavioural data showed contrast effects with large inducers and assimilation effects with small inducers, as expected. The contrast responses were faster and stronger with white than with black inducers, whereas assimilation responses were faster and stronger with black than with white inducers. Specifically, there were significantly more assimilation responses than contrast responses with BS inducers, and significantly more contrast responses than assimilation responses with WL inducers. In the other two conditions (BL and WS) the responses were a mixture of contrast and assimilation. This was somewhat unexpected as it is generally reported that these stimuli produce more consistent contrast and assimilation responses, respectively (e.g., Beck 1966; Helson 1963; Soranzo et al. 2010; Wade 1996). However, it is consistent with the idea that contrast is stronger in the WL than BL condition and assimilation is stronger in the BS than WS condition, because in the conditions, where the effect is thought to be weaker, participants may have given mixed responses due to uncertainty around a less obvious perceived difference between the target and comparison square.

The finding that the contrast effects were stronger with the white than the black inducers is inconsistent with the predictions of Anchoring Theory (Gilchrist et al. 1999) and with some previous reports, stating that the simultaneous contrast illusion arises primarily as a result of a contrast effect on the target surrounded by black (Economou et al. 2007). This difference could result from a difference in stimulus design: Economou et al. (2007) presented a ‘traditional’ simultaneous lightness contrast display (two grey targets, one surrounded by white and one surrounded by black), whereas the current work presented only ‘half’ of this display at a time (i.e., either black or white inducer, not both), thereby changing/removing the ‘global framework’. However, others have reported a stronger contrast effect when a grey disc was grouped with the white inducers than the black inducers (Murgia et al. 2016), which suggests that the Anchoring Theory does not predict the strength of contrast effects in all cases.

The finding that the assimilation effects were stronger with the black than the white inducers is consistent with the findings of Soranzo et al. (2010). It is also consistent with Murgia et al.’s (2016) finding of a stronger assimilation effect when a grey target was intentionally grouped with the black inducers than the white inducers. Beck (1966) and de Weert and Spillman (1995) also reported that assimilation occurred with black inducers while contrast occurred with white inducers, which is consistent with the results of the forced-choice task, where some participants giving contrast responses in the WS condition and others giving assimilation responses, whereas responses in the BS condition were more consistently assimilation responses.

The results pertaining to the asymmetry in contrast and assimilation with black or white inducers may be explained by the luminance ratio between the target and inducer. For example, Rudd and Zemach (2004, 2005) measured the relative induction strengths of white and black inducers with classical disk/annulus stimuli and found that contrast effects were stronger with black inducers than with white inducers. It should be noted that lightness contrast depends on the luminance ratio between the target and its surround (Rudd and Zemach 2005). In the current study’s stimuli, the luminance ratio between the target and the black inducers was 54.3 cd/m^2^ (29.89/0.55). However, this ratio was only 3.2 cd/m^2^ when the inducers were white (95.78/29.89). These physical characteristics of the display can potentially explain the asymmetries which emerged in our study with different inducers and explain the apparent contradiction with previous literature. Comparing our results with previous literature, it can be concluded that while black inducers might generate stronger contrast effects, the magnitude of the effect largely depends on the luminance ratio between target and inducers, and this would be interesting to explore with a greater range of luminance ratios in future research. The edge integration theory (Rudd 2010, 2017) may be a more suitable model to account for the asymmetry and proposes that assimilation requires a higher level of cortical processing as well as contrast.

Although there are some inconsistencies between the current research and previous literature with regard to the contrast effect being stronger with the white inducer, these would be explained if contrast and assimilation are to be conceptualised as opposing processes: one effect is strengthened in conditions with white inducers, whereas the other is strengthened in conditions with black inducers. It should be noted that the inducer colour alone is not being regarded as a determining factor for whether a stimulus elicits a contrast or an assimilation response, but that it is important in conjunction with the configuration of the inducers. It could be argued that the configuration and colour of the inducers are both contributing factors when making judgements about the salience of each surface within an image. De Weert and Spillman (1995) suggested that assimilation effects with inducers that were darker than the target could be a result of the dark surfaces being perceived as ‘figure’ and the areas bordering the dark surfaces (i.e., the grey target) being perceived as ‘ground’. This suggestion hints at the possibility that the colour and configuration of a stimulus can affect mediating processes, such as figure/ground segregation, which could subsequently alter the likelihood of a contrast vs assimilation effect for the stimulus concerned. In the context of the current results, the combination between the large and the white inducers may be the most likely pairing to result in a figure-ground interpretation that favours contrast, whereas the combination between small and black inducers may be the most likely pairing to result in a figure-ground interpretation that favours assimilation. Future research should consider ways to assess participants’ interpretation of figure and ground within the stimuli to fully investigate the relationship between figure-ground interpretation and the strength of contrast/assimilation effects, including underlying ERP differences.

Unlike the strength of effects, response time relating to contrast and assimilation has not been previously reported in literature. This is likely due to the constraints of task type/paradigm. For example, participants are usually given an unlimited amount of time to make a lightness judgement, and the speed of response is not recorded, whereas the forced choice task used in this ERP study allows easy measurement of the speed of responses. RT analysis showed that the conditions with faster RTs were the same that showed stronger contrast and assimilation responses as determined by the consistency of responses, i.e., the WL and BS conditions, respectively. The conditions with slower overall RTs, namely, the BL and WS conditions, were also the conditions which showed a mixture of contrast and assimilation responses rather than a clear majority of either response type.

Although our experiment was not a typical speeded task, it is interesting to note that contrast responses were faster in the WL condition than in the BL and assimilation responses were faster in the BS than the WS conditions. This may suggest that the contrast effect is more obvious in the WL condition and assimilation is more obvious in the BS condition. This suggestion is based on the assumption that participants take longer to decide in conditions, where the effect is not apparent, even if they are not requested to complete the task too quickly. Our suggestion is supported also by the number of responses of each type per condition. The number of responses per condition represents the strength of the contrast or assimilation. The coherence between the RTs and the number of responses reinforces the assumption that these measures may be used to indicate the strength of the contrast and assimilation. Moreover, the RT measures were also well aligned with the ERP measures (see below), further supporting the suggestion that contrast is more obvious in WL condition and assimilation—in the BS condition.

### ERP data (Hypotheses 2–4)

The ERP results showed greater N1 amplitude for assimilation than for contrast responses in the left occipital area, in the WS condition. Across contrast and assimilation responses, the parietal area showed greater amplitude and longer latencies than the occipital and occipital–parietal areas. Given that contrast and assimilation are both lightness-related effects, some level of similarity in the underlying processing is to be expected, however, it was of interest to attempt to discover potential sources of difference between the two effects, particularly as they can be thought of as operating in opposite directions (i.e., contrast makes targets appear more different to their inducers, whereas assimilation makes targets appear more similar to their inducers). In previous research, an increase in N1 amplitude has been associated with enhanced processing and attention selection, suggesting a potential role for a higher level of selective attention to a less obvious assimilation effect in WS condition (as compared to stronger assimilation effect in BS condition). This effect was also accompanied by smaller number and slower RTs of responses to assimilation than to contrast. An effect at the time period of N1 suggests that there is a difference in processing of contrast and assimilation at a higher cortical level, as N1 is thought to have extrastriate origins (Gomez-Gonzales et al. 1994). This difference could also result from a difference between contrast and assimilation in terms of the influence of higher level processing such as the global perceptual organisation of the stimulus (e.g., the ‘frameworks’ or figure-ground segmentation implied by the stimulus), as this information can be fed back to V1 from ~ 100 ms (Lamme and Roelfsema 2000).

With the exception of the difference in N1 amplitude, there were no other effects concerning response type (contrast vs assimilation) on ERP measures. The general lack of significant differences in ERP measures between contrast responses and assimilation responses when all else is held constant suggests that contrast vs assimilation responses are largely not associated with different electrophysiological processing in the occipital and parietal regions in the time windows up to 300 ms following stimulus onset. It could be assumed that a null result suggests that similar processing is involved with both contrast and assimilation; however, Otten and Rugg (2005) outline several reasons why this conclusion would not necessarily be correct. The most important of these is that ERPs reflect only the brain activity which is detectable by scalp surface electrodes; typically, synchronous activity from populations of cortical pyramidal neurons oriented perpendicular to the electrode positions (Jackson and Bolger 2014), and the neural activity which differentiates two response types may not have the properties necessary for detection by electrodes on the scalp. Therefore, it is not possible to conclude that a non-significant difference implies entirely equivalent neural processing.

When considering only contrast responses, the P1 amplitude was larger for contrast responses with black inducers than with white inducers in the right occipital–parietal area. Similarly to the N1 amplitude, this effect was accompanied with slower RTs and a weaker contrast effect (i.e., smaller number of contrast responses) with the black inducers. These findings indicate that black inducer condition had the less obvious contrast effect and could be regarded as the more ‘difficult’ of these two conditions, and the larger amplitude suggests that it may require more processing in the right occipital area. The difference in P1 amplitude could reflect a difference in the amount of processing given to the stimulus, rather than simply luminance-based processing which occurs at a retinal level. Given that white inducers favour a stronger contrast effect and a lower amplitude of neural activity, it is possible that the decision about the target lightness has been made more quickly and with less processing resources than with black inducers. One other explanation for this difference in P1 amplitude could simply be that this reflects the difference in the luminance ratios of the black and white inducers.

Consistently with the two previous results discussed above, the N2 amplitude appeared to be larger for assimilation responses in the ‘more difficult’ condition with white inducers as compared with black inducers in both hemispheres. This result was not statistically significant; however, this effect too was accompanied by fewer and delayed responses to the WS condition. The pattern of these results indicates that perception of lightness contrast and assimilation is a process accomplished at a cortical level and may closely correlate with the behavioural performance, whereby weaker effects need more processing time and greater neural activity in occipital sites, either right or left, whereas overall activation for both contrast and assimilation was larger in the right parietal areas.

Thus, the findings from the current study suggest that contrast may occur very early and around 110 ms and it needs more processing both behaviourally and in terms of brain activation in the BL condition compared to the WL condition (WL has the stronger contrast effect). The assimilation difference between BS and less obvious WS condition occurred around 220 ms, according to our ERP results, which could be an indicator of the processing time required for this complex perceptual phenomenon. Therefore, our results suggest the following time course of brain activation to lightness perception: the predominant activity associated with the strength of a contrast effect occurs around 110 ms and that associated with the strength of an assimilation effect occurs around 220 ms, while there is sometimes a difference in activity between contrast vs assimilation responses to the same physical stimulus around 170 ms.

Theoretical implications from the current research corroborate previous suggestions of contrast and assimilation being processed at the cortical level, i.e., in parietal and occipital areas, in addition to the retinal processing. Current theories should account for the ‘asymmetry’ associated with inducer colour, i.e., explain why white inducers favour a stronger contrast effect, while black inducers favour a stronger assimilation effect. This could be linked to the role of figure-ground or attentional salience or weighting of different surfaces or ‘frameworks’. Finally, theories need to give scope for explaining contrast and assimilation as part of a continuum of effects or explaining the shift between contrast and assimilation in a way which is not only reliant on spatial frequency. This would take into account the finding that sometimes, particularly in a forced-choice task, an identical stimulus can result in either a contrast or an assimilation response from different individuals, or indeed on different occasions for the same individual. The finding that activity in the right occipital–parietal area (P1 time window) was greater to the weaker (BL) contrast effects, and the N1 was greater to weaker assimilation than contrast effect in the left occipital–parietal area, also imply possible neural markers for a continuous variation in the strength or type of perceived effect. We should, however, take into account that quasi-experimental approach used in our study, i.e., assigning participants to groups based on their perceptual responses, could be a limitation and our findings need to be interpreted with caution until replicated in future research.

In conclusion, the current work provided further evidence to support cortical involvement rather than retinal processing alone for both contrast and assimilation as an ongoing electrophysiological activity was recorded in the occipital and parietal cortex, implying processing at visual cortical areas, such as V1, and beyond. The difference at the N1 measurement window in WS condition also suggested a point of divergence between the two phenomena at a cortical level, while contrast occurs earlier than assimilation.

## Data Availability

The materials and data used for the writing up of this publication are available and can be provided on request.
